# Ancient Chinese Herbal Recipe Huanglian Jie Du Decoction for Ischemic Stroke: An Overview of Current Evidence

**DOI:** 10.14336/AD.2022.0311

**Published:** 2022-12-01

**Authors:** Chao-Chao Yu, Le-Bin Liu, Shi-Yuan Chen, Xiao-Fei Wang, Li Wang, Yan-Jun Du

**Affiliations:** ^1^The Fourth Clinical Medical College of Guangzhou University of Chinese Medicine, Shenzhen, Guangdong, China.; ^2^Department of Integrated Chinese and Western Medicine, Zhongnan Hospital of Wuhan University, Wuhan University, Wuhan, Hubei, China.; ^3^Department of Rehabilitation Medicine, Hubei Rongjun Hospital, Wuhan, Hubei, China.; ^4^Department of Rehabilitation Medicine, Wuhan Third Hospital, Tongren Hospital of Wuhan University, Wuhan, Hubei, China.; ^5^College of Acupuncture and Orthopedics, Hubei University of Chinese Medicine, Wuhan, Hubei, China.

**Keywords:** ischemic stroke, Huanglian Jie Du decoction, berberine, baicalin, geniposide

## Abstract

Ischemic stroke is a major cause of mortality and neurological morbidity worldwide. The underlying pathophysiology of ischemic stroke is highly complicated and correlates with various pathological processes, including neuroinflammation, oxidative stress injury, altered cell apoptosis and autophagy, excitotoxicity, and acidosis. The current treatment for ischemic stroke is limited to thrombolytic therapy such as recombinant tissue plasminogen activator. However, tissue plasminogen activator is limited by a very narrow therapeutic time window (<4.5 hours), selective efficacy, and hemorrhagic complication. Hence, the development of novel therapies to prevent ischemic damage to the brain is urgent. Chinese herbal medicine has a long history in treating stroke and its sequela. In the past decades, extensive studies have focused on the neuroprotective effects of Huanglian Jie Du decoction (HLJDD), an ancient and classical Chinese herbal formula that can treat a wide spectrum of disorders including ischemic stroke. In this review, the current evidence of HLJDD and its bioactive components for ischemic stroke is comprehensively reviewed, and their potential application directions in ischemic stroke management are discussed.

## 1. Introduction

Stroke, which can be classified as ischemic or hemorrhagic, is the third leading cause of death worldwide following cancer and coronary heart disease and leads to permanent disability in 80% of convalescents [1, 2]. The ischemic stroke accounts for approximately 70% of all stroke types, with the remaining subtypes being intracerebral hemorrhage or subarachnoid hemorrhage [1]. Ischemic stroke is caused by thrombosis or embolism in cerebral vessels responsible for blood supply to functional areas of the brain. Currently, intravenous thrombolysis and endovascular clot retrieval, which can remove the obstruction and restore blood flow, have been regarded as effective treatments for cerebral ischemia [3, 4]. Nevertheless, the following loss of neurologic function remains difficult to solve during post-stroke rehabilitation. Cascaded metabolic and cellular damages will occur after cerebral ischemia, including neuroinflammation [5], oxidative stress damage [6], alternated autophagy and apoptosis [7, 8], excitotoxicity [9], and dysregulated angiogenesis [10]. It is indicated that agents for multiple events occurring in cerebral ischemia could be a rational and promising direction [11].

Traditional Chinese medicine (TCM) therapies have been practiced in China for more than 2000 years. Among them, Chinese herbal medicine and acupuncture are the most common treatments for stroke and post-stroke disabilities. Huanglian Jie Du Decoction (HLJDD, Oren-gedoku-to in Japanese), composed of Rhizoma coptidis, Radix scutellariae, Cortex phellodendri, and Fructus gardeniae at a ratio of 3:2:2:3, is a classical and famous multi-herb remedy recorded in *Wai Tai Mi Yao* in the Tang dynasty (752 A.D.). *Wai Tai Mi Yao* is a well-known classic TCM monograph for heat-clearing and detoxicating. The herbs in HLJDD are officially listed in the Chinese pharmacopeia. In recent years, increasing evidence has revealed the pharmacological effects of HLJDD on gastrointestinal disorders [12], diabetes [13, 14], Alzheimer’s disease [15], depression [16], cancers [17, 18], and cardiovascular diseases [19, 20]. In addition, substantial scientific evidence has suggested that HLJDD exhibits significant neuroprotective effects during stroke treatment. To the best of our knowledge, there has been no systematic summary of the neuroprotective effects of HLJDD on ischemic stroke. This review aims to systematically summarize the current evidence of the pharmacological mechanism of HLJDD in ischemic stroke and discuss its future application directions in stroke treatment.

**Figure 1.**
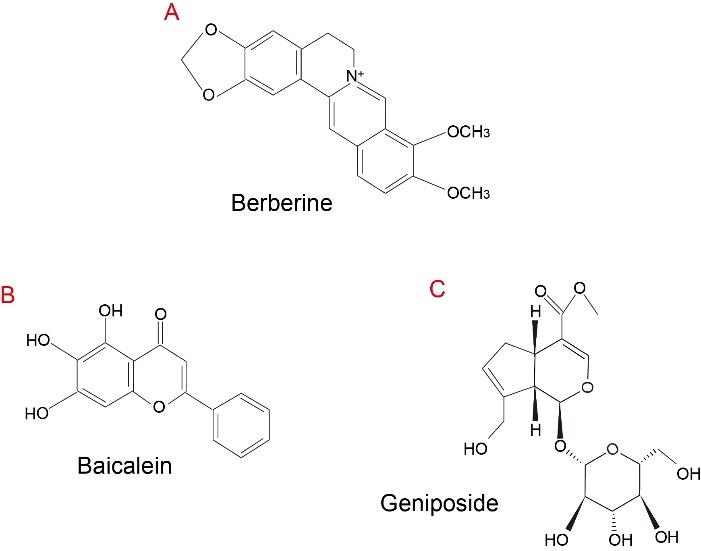
The chemical structure of berberine, baicalin, and geniposide.

## 2. Phytochemical and Pharmacokinetic Studies on HLJDD in Ischemic Stroke

Though HLJDD has been widely used in treating a wide spectrum of disorders, its bioactive components and metabolites are poorly understood. Plasma pharmaco-chemical approaches are increasingly applied in the identification of bioactive ingredients in TCM [21]. According to plasma pharmacochemistry, only the constituents absorbed into the blood could yield pharmacological effects and may be regarded as potential bioactive constituents. Transcriptomics analysis and metabonomics analysis by the high-performance liquid chromatographic method are usually conducted to identify active components of HLJDD. In recent years, growing evidence has demonstrated that alkaloids (e.g., berberine, palmatine, and coptisine) from Rhizoma coptidis and Cortex phellodendri, flavonoids (e.g., baicalin, baicalein, wogonoside, and wogonin) from Radix scutellariae, and iridoids (e.g., geniposide and shanzhiside) from Fructus gardeniae are major bioactive ingredients in HLJDD. Among them, berberine, baicalin, and geniposide exert the most significant pharmacological effects (Fig. 1). Therefore, they are defined as quality control markers for HLJDD [22-29]. Metabolic pathway analysis of HLJDD in rats using liquid chromatography-ion trap mass (LC-IT-MS) combined with liquid chromatography-Fourier transform ion cyclotron resonance mass spectrometry (LC-FT-ICR-MS) indicated that glucuronidation and sulfation reactions are the primary biotransformation pathways in plasma; additionally, the hydroxylation, demethylation, and glucuronidation of alkaloids and glucuronidation and sulfonation of both iridoids and flavonoids are the main metabolic pathways existed in urine and feces [30]. Serum pharmacokinetics analysis of berberine derived from HLJDD by high-performance liquid chromatography-tandem mass spectrometry (LC-MS-MS) suggested that T_max_ is approximately 54 minutes, and T_1/2_ is 218.353 minutes [31]. Additionally, co-administration of geniposide with baicalin significantly increased the bioavailability of geniposide in a rat model of cerebral ischemia. However, berberine showed no significant effects on the pharmacokinetics of geniposide and decreased the bioavailability of geniposide co-administrated with baicalin [32]. This difference can be attributed to baicalin neutralization and intensified geniposide hydrolysis induced by berberine. Zhu et al. investigated the plasma concentration-time profiles and distribution characteristics of the bioactive components derived from HLJDD in middle cerebral artery occlusion (MCAO) rat tissues. It was found that berberine, palmatine, baicalin, and baicalein dispersed rapidly and a large amount of them accumulated in the lung tissue, and gardenoside displayed the highest bioavailability in the lung and kidney [33]. This result suggests pharmacological effects of HLJDD and other medications and potential drug interactions in vivo in different target tissues. A ^1^H NMR-based serum and brain metabolomics study revealed that refined HLJDD treatment within 5 h exhibit significantly neuroprotective effects on cerebral ischemia-reperfusion injury by ameliorating the oxidative stress status, blocking the inflammatory cascade, alleviating the malfunction of the energy metabolism, and reversing altered amino acid and nucleic acid metabolisms [34].

When orally administrated, Chinese herbal medicine undergoes a series of complex pharmacokinetic processes, including intestinal absorption, circulatory distribution, hepatic metabolism, and renal excretion. Prototype components of Chinese herbal medicine are delivered to various lesions after entering the blood directly or metabolized into other substances or excreted. However, multi-component herbal medicines are more complicated than compounds with a single active ingredient. Furthermore, the prototypical components of herbal medicines and their metabolites are metabolized by symbiotic intestinal microorganisms and various hepatic drug enzymes in vivo, which in turn changes multiple metabolic profiles, for example, endogenous small molecule metabolic profiles and intestinal microbial metabolites. Before the activity evaluation of the components of western botanical medicine, every single compound needs to be separated. However, if the components of traditional Chinese herbal medicine are separated or isolated, the multi-component, multi-target, and multi-pathway mechanism cannot be presented and therefore, the advantages of syndrome differentiation and treatment cannot be exhibited. In fact, the overall efficacy of traditional Chinese herbal medicine usually depends on the combined effect of pharmacodynamic substances in the compound. The content of HLJDD cannot be elucidated by a single compound phytopharmaceutical research method. Therefore, an integrated pharmacokinetic and metabolomic approach is needed to reveal the pharmacodynamic substance basis of HLJDD [35].

## 3. Effects of HLJDD and Its Bioactive Components on the Neuropathology of Ischemic Stroke

### 3.1 Anti-Apoptosis

Substantial evidence suggests that cell apoptosis plays a significant role in neuronal death after cerebral ischemia/reperfusion (I/R) injury [36, 37]. Both extrinsic (e.g., death-receptor-mediated apoptosis) and intrinsic apoptotic pathways (e.g., mitochondria-mediated apoptosis, calpain-mediated apoptosis, p53-mediated apoptosis, p38-mediated apoptosis, ERK-mediated apoptosis, JNK-mediated apoptosis, and endoplasmic reticulum-mediated apoptosis) play a critical role in recruiting downstream apoptotic molecules to trigger cell death [38]. Caspases and Bcl-2 family members are crucial regulators of multiple apoptotic signal transductions [39, 40]. Among caspases, caspase-8, caspase-9, and caspase-10 are initiators of apoptotic caspases, and caspase-3 and caspase-7 are executors. In addition, caspase-3 and caspase-9 are associated with apoptosis that leads to neuronal death. The Bcl-2 family includes anti-apoptotic (e.g., Bcl-2 and Bcl-xL) and pro-apoptotic proteins (e.g., Bax) [41, 42]. Overwhelming evidence supports those active ingredients derived from HLJDD could protect against cerebral ischemia via anti-apoptosis effects. Zheng et al. [43] reported that baicalin could significantly alleviate neurological deficits, reduce infarct volume in a rat model of middle cerebral artery occlusion (MCAO)/reperfusion, and inhibit ischemia/hypoxia-induced neuronal apoptosis by increasing Bcl-2 expression. These effects are attributed to myocardin-related transcription factor-A(MRTF-A)-mediated transactivation in phosphatidylinositol-3kinase (PI3K) and extracellular signal regulated kinase-1/2 (ERK1/2) pathways. Activation of the PI3K/Akt signaling pathway contributes to the amelioration of hypoxic-ischemic-induced neuronal injury, and LY294002, an inhibitor of PI3K, reverses baicalin-induced anti-apoptosis effects [44]. Similarly, berberine can also inhibit cell apoptosis through activating the brain-derived neurotrophic factor (BDNF)-TrkB-PI3K/Akt pathway in a rat model of MCAO [45]. In addition, berberine can reverse oxygen-glucose deprivation/reperfusion-induced apoptosis in PC12 cells, which is associated with inhibition of endoplasmic reticulum (ER)-mediated apoptosis and indicated by decreased endoplasmic reticulum stress-related markers, including glucose-regulated protein 78 and C/EBP homologous protein [46]. Furthermore, Zhao et al. demonstrated that berberine inhibits endoplasmic reticulum-mediated apoptosis in rats with cerebral ischemia-reperfusion injury by downregulating the canopy FGF signaling regulator 2 (CNPY2)/double-stranded RNA-activated protein kinase-like ER kinase (PERK) signaling pathway [47]. Additionally, berberine-mediated neuroprotection after cerebral ischemia involves the activation of Akt/GSK3β and ERK 1/2 pathways [48] and downregulation of p53 [49] in cells exposed to oxygen and glucose deprivation and AMPK in vivo [50]. These studies indicate that the bioactive components of HLJDD can inhibit cerebral ischemia-induced neuronal apoptosis via multiple pathways, thereby exerting a neuroprotective effect.

### 3.2 Anti-Oxidative Stress

Free radicals, including reactive oxygen species (ROS), superoxide anion (O^2-^), hydroxyl radicals (OH^-^), and hydrogen peroxide (H_2_O_2_), and reactive nitrogen species (RNS) which can be further divided into NO and ONOO^-^, are implicated in ischemia-reperfusion injury [51, 52]. Oxidative stress is caused by excess production of ROS via the mitochondrial respiratory chain, NADPH oxidases, the reaction of arachidonic acid catalyzed by cyclooxygenase 2, and oxidation of xanthine and hypoxanthine by xanthine oxidase [53]. Accumulating evidence has supported that oxidative stress can cause cell death through DNA damage [54] and lipid peroxidation [55]. NO is generated from endothelial NOS (eNOS), neuronal NOS (nNOS), and inducible NOS (iNOS) [56, 57]. The overproduction of NO can lead to blood-brain barrier (BBB) disruption [58], cell death [59], and inflammation [60]. Yuan et al. showed that HLJDD and its major bioactive components could prevent cerebral microvascular endothelial cells from hypoxia and reoxygenation injury [61]. Zhang et al. reported that HLJDD increases levels of antioxidant enzymes in MCAO rats, including superoxide dismutase (SOD), glutathione peroxidase (GPx), and HO-1 that scavenges ROS during ischemia-reperfusion by activating nuclear erythroid 2-related factor 2 (Nrf2), a transcription factor that stimulates ROS detoxification and the expression of various antioxidant genes including HO-1 [62]. In addition, baicalin exerts significant anti-oxidative stress effects via activating the BDNF/TrkB/PI3K/AKT pathway and BDNF/TrkB/MAPK/ERK pathway in neuron-astrocyte cocultures exposed to oxygen-glucose deprivation/reoxygenation [63]. Li et al. proved that baicalin prevents mitochondrial fission and excessive production of ROS by activating AMPK [64]. Moreover, baicalin can inhibit mitochondrial succinate dehydrogenase (SDH) from reducing the production of ROS and prevent the subsequent loss of glutamine synthetase (GS) sensitive to oxidative stress in vivo and in vitro, thus enhancing glutamate disposal by astrocytes and decreasing excitotoxicity [65].

### 3.3 Anti-Neuroinflammation

Secondary neuroinflammation following ischemic stroke causes further injury [66]. Cerebral ischemia/reperfusion injury can activate microglia, astrocytes, and resident immune cells and influence the infiltration of circulating immune cells, including neutrophils, monocytes, blood-derived macrophages, and T cells into the ischemic lesions. These neuroinflammatory events and following apoptosis and oxidative stress can lead to cell death and thus aggravate brain damage [67, 68]. Neuroinflammation has become a target for therapeutic intervention [66]. Recently, pharmacology and molecular docking analyses revealed that HLJDD and its bioactive compounds (baicalin and geniposide) exert anti-inflammatory effects by inhibiting the 5-lipoxygenase (5-LOX)/CysLTs pathway in ischemic stroke [69, 70]. Hwang et al. reported that HLJDD and its bioactive ingredient baicalein reduce neutrophil infiltration in ischemic brain tissue by up to approximately 30% in MCAO rats [71]. It has been suggested that aqueous extract and major components (alkaloids, flavonoids, and iridoid) of HLJDD prevent abnormal activation of astrocytes after ischemic stroke in MCAO rats, as evidenced by decreased levels of glial fibrillary acidic protein and connexin43 [72]. A clinical trial indicated that berberine treatment could alleviate carotid atherosclerotic plaque, decrease the level of serum inflammatory cytokines, including IL-6 and migration inhibitory factor, and improve the prognosis of patients with acute ischemic stroke [73]. In addition, berberine can lower proinflammatory cytokines such as TNF-α and IL-1β in rats with focal cerebral ischemia [74]. Several signaling pathways participate in the anti-inflammatory effects of berberine, baicalin, and geniposide. For example, berberine inhibited the high-mobility group box1 (HMGB1) protein/toll-like receptor 4 (TLR4)/nuclear factor kappa B (NF-κB) pathway [ 75, 76] and inactivated NLRP3 inflammasome through activating AMPK signaling pathway [77, 78]. Besides, berberine activated peroxisome proliferator-activated receptor-gamma (PPARγ) [79]. Berberine and baicalin also inhibited the NF-κB/CCL2/CCTR2 pathway [80] via promoting microglia M2 polarization and inhibiting M1 polarization through activating AMPK [81]. Additionally, baicalin alleviates the loss of dendritic spines and relieves cognitive decline in mice with repeated cerebral ischemia-reperfusion injury by remodeling the gut microbial composition to decrease the hippocampal proinflammatory cytokines, including IL-1β, IL-6, and TNF-α [82]. Results from another ^1^H NMR metabolomics study further validated that berberine, baicalin, and geniposide isolated from HLJDD inhibit neuronal autophagy and inflammation by activating the AKT/GSK3β signaling pathway and inhibiting nuclear translocation of NF-κB-p65 and overexpression of proinflammatory genes (TNF-α, IL-1β, IL-2, and IL-6) and enzymes (iNOS and COX-2) [34]. These studies suggest that HLJDD and its bioactive ingredients have a promising potential to be applied in stroke treatment. Cholinergic, purinergic, and glutamatergic receptors and transporters are also closely associated with the inflammatory response following stroke [83]. However, the effects of HLJDD on these neurotransmission signalings have been barely investigated in animal models of stroke. Moreover, a variety of studies demonstrated that infiltration of leukocytes, neutrophils, blood-derived macrophages, and T lymphocytes is involved in the pathogenesis of cerebral ischemia. These peripheral immune cells can promote the production of proinflammatory factors and drive secondary neurodegeneration after ischemic stroke [84, 85]. At present, the potential impacts of HLJDD on these circulating immune cells have not been elucidated in detail.

### 3.4 Anti-Calcium Overload

Calcium overload has been reported to be a major cause of neuron death and brain damage following ischemic stroke [86]. Ca^2+^ ions are substantial for the survival and functioning of neurons and neuroglia [87]. The increased Ca^2+^ influx and impaired Ca^2+^ extrusion across the plasma membrane led to intracellular Ca^2+^ accumulation, which triggers various harmful processes such as free radical production [88], mitochondrial dysfunction [89], inflammation [90], and DNA damage and thus leads to irreversible cellular damages, ultimately causing apoptotic and necrotic death [91]. HLJDD has been reported to exert antagonistic effects on NMDA receptor-dependent calcium channel opening, voltage-gated calcium channel opening, external calcium inward flow induced by calcium carrier loading, calcium pool release regulated by the IP_3_ (inositol triphosphate) receptor system, and the Ryanodine receptor system in hypoxia/hypoglycemia-induced cerebral ischemia-like neurons [92]. Nadjafi et al. reported that berberine treatment at 2 μM significantly increases oligodendrocyte viability under the oxygen-glucose deprivation/24 h reperfusion condition associated with the prevention of intracellular calcium accumulation [93]. Similarly, another bioactive component derived from HLJDD baicalin also alleviates oxygen-glucose deprivation-induced human brain microvascular endothelial cell apoptosis by inhibiting intracellular calcium overload. This effect is partly due to the upregulation of Na^(+)^-K^(+)^-ATPase and Ca^(2+)-^ATPase [94].

### 3.5 Anti-Neural Injury

Mounting evidence has proved that HLJDD exerts neuroprotective effects against ischemia/reperfusion injury through multiple pathways, including alleviating neuronal loss in MCAO rats [95], prompting axon repair by inhibiting NogoA/NgR after focal cerebral ischemia in mice [96], and increasing acetylcholine content in rats with cerebral ischemia [97]. However, effects of HLJDD on other specific neuropathologies after ischemic stroke, including impaired synaptic plasticity, axonal transport dysfunction, altered neurotransmitters release, are poorly understood. Synaptic and mitochondrial dysfunction plays a crucial role in the pathophysiological process of neural damage after cerebral ischemia [98-100]. It has been demonstrated that berberine and geniposide can alleviate axonal mitochondrial abnormalities and synaptic dysfunction [101, 102]. In addition, berberine can inhibit glutamate release via the suppression of presynaptic Cav2.1 channels and ERK/synapsin I signaling cascade to reduce glutamate excitotoxicity [103]. The glutamate excitotoxicity occurs early after the ischemic insult and is a key target for increasing neuron survival [52].

### 3.6 Pro-Neurogenesis

Neurogenesis is known to be induced in infarcts and surrounding lesions in response to ischemic stroke [104]. Neural stem cells originating from the subventricular zone of the lateral ventricle and the dentate gyrus of the hippocampus [105] can be activated after ischemic stroke. Additionally, they can proliferate and differentiate into new neurons and migrate to the ischemic lesion [106], which is considered a vital process in post-stroke recovery and repair of the injured brain [107]. A variety of growth factors, including brain-derived neurotrophic factor (BDNF), neurotrophin-3 fibroblast growth factor (FGF), transforming growth factor (TGF)-α, TGF-β, epidermal growth factor (EGF), vascular endothelial growth factor (VEGF), and insulin-like growth factor-1 (IGF-1), have been identified to enhance neurogenesis and angiogenesis in response to stroke [107]. Moreover, the Notch pathway, PI3K signaling pathway [108], BDNF signaling pathway [109], Wnt/beta-catenin pathway [110], and Sonic hedgehog pathway [111] are also associated with neurogenesis in ischemic stroke. Zou et al. reported that alkaloids, flavonoids, and iridoids from HLJDD enhance neurogenesis in rats subjected to MCAO, which is attributed to the activation of Akt/GSK-3β signaling, upregulation of VEGF, angiopoietin-1, and angiopoietin-2 [112]. Additionally, HLJDD can activate the PI3K/Akt pathway and hypoxia-inducible factor-1 (HIF-1) in animals with cerebral ischemia [113, 114], a transcription factor mediating ischemic tolerance through increasing glucose uptake and glycolysis and activate vasculogenesis genes such as erythropoietin (EPO) and VEGF [115]. However, existing studies of the effects of HLJDD and its bioactive components on neurogenesis in ischemic stroke lack comprehensiveness. The underlying mechanisms of HLJDD -induced pro-neurogenesis need further investigation.

### 3.7 BBB Protection

The BBB, which plays a fundamental role in maintaining central nervous system homeostasis, is made up of endothelial cells and tight junctions, pericytes, astrocytic end-feet, and extracellular matrix components [116-118]. Post-stroke BBB breakdown, characterized by structural disruption of tight junctions and increased permeability, results in the infiltration of peripheral immune cells and metabolic compounds into the brain and their accumulation in the brain. The infiltration and accumulation are prominent pathological features of neurodegenerative disease [119] and stroke [120] and are usually associated with a poor prognosis for stroke [121]. Therefore, it is significant to identify promising potential therapeutic targets for protecting the BBB so as to promote post-stroke recovery [122]. It has been demonstrated that baicalin can reduce MCAO-induced BBB permeability increase and brain edema by inhibiting matrix metalloproteinase-9 (MMP-9) in rats [123] and preventing the loss of tight junction proteins zonula occludens-1 and claudin-5 in microvascular endothelial cells [124, 125]. Matrix metalloproteinases (MMPs) belong to zinc-endopeptidases [126]. Zinc accumulation in microvessels after ischemic stroke activates MMP-9 and MMP-2, leading to occludin and claudin-5 loss and tight junction protein degradation and then BBB damage [127]. In addition, Li et al. indicated that geniposide restores BBB integrity by promoting expression levels of zonula occludens-1 and claudin-5 and downregulating the expression of MMP-9 and MMP-2 in vitro BBB model consisting of primary cultures of brain microvascular endothelial cells and astrocytes [128]. Hemorrhagic transformation is a frequent and generally asymptomatic change that occurs after acute ischemic stroke and is caused by the extravasation of blood products due to abnormal BBB permeability into the infarcted brain area [129]. Hemorrhagic transformation can cause clinical deterioration and poor outcomes [130]. Therefore, antithrombotic and thrombolytic drugs for preventing blood coagulation and fibrinolysis have been used [130]. Kim et al. reported that HLJDD showed potent inhibitory effects on platelet aggregation and thrombus formation via inhibiting the phosphorylation of phospholipase C and protein kinase B in mice [131]. It has been validated that HLJDD can inhibit platelet aggregation and thrombosis in patients with cerebral infarction [132] and the rat model of MCAO [133].

## 4. Discussion

**Figure 2.**
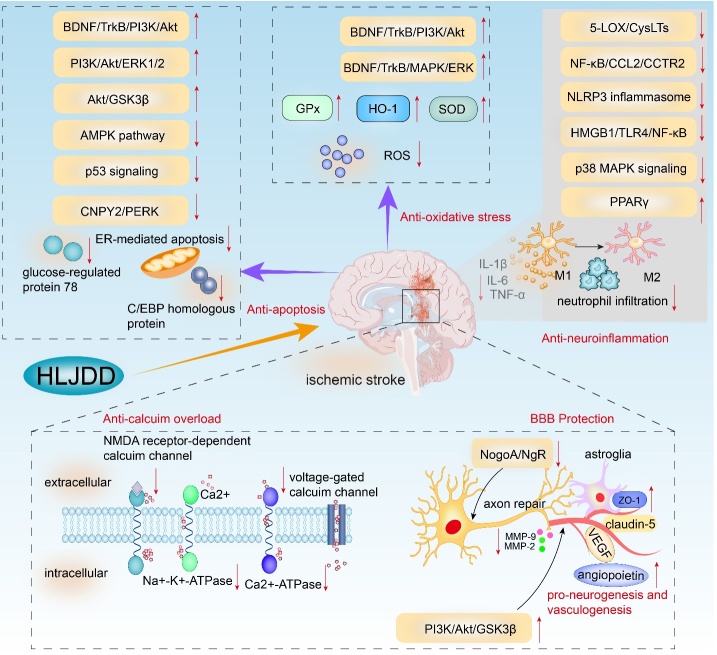
Schematic representation of neuroprotective effects of HLJDD in ischemic stroke. The red downward arrow represents inhibitory effects, while the red upward arrow represents stimulative effects.

It has been found that HLJDD exerts neuroprotective effects against ischemic stroke through multiple mechanisms, including anti-apoptosis, anti-inflammation, anti-oxidative stress, anti-calcium overload, anti-neural injury, blood-brain barrier protection, and pro-neurogenesis (Fig. 2). Nevertheless, previous studies have several limitations. First, the existing experimental studies on potential neuroprotective mechanisms are not comprehensive or in-depth. How HLJDD influences the extrinsic or intrinsic pathway or other crucial regulators of apoptotic pathways to prevent neuronal apoptosis has not been fully addressed.

Growth factors or vital signaling pathways involved in prompting neurogenesis and angiogenesis have rarely been investigated. Additionally, effects of HLJDD on the key components of the blood-brain barrier such as endothelial cells, pericytes, astrocytes, and extracellular matrix components are barely studied and only several tight junction proteins and MMPs are observed. In order to evaluate the effects of HLJDD on ischemic stroke more objectively, non-invasive molecular imaging modalities, such as magnetic resonance imaging (MRI) and positron emission tomography (PET), should be applied in future studies to assess BBB structure and function. Second, more attention should be paid to whether HLJDD could ameliorate neuropsychiatric alternations, such as post-stroke depression and cognitive disorder. Ye et al. demonstrated that HLJDD alleviated depression-like behaviors in rats with chronic unpredictable stress through activating the BDNF-TrkB-CREB pathway [134]. A recent study combining network pharmacology and metabolomics analysis also revealed that HLJDD exerted antidepressant effect via potentially targeting serotoninergic and dopaminergic synaptic functions and several signaling pathways such as PI3K/Akt pathway and mTOR signaling pathway [16]. Anti-depressive effects of HLJDD in post-stroke should be validated in the future. Third, genes, proteins, metabolites, and signaling pathways involved in the neuroprotective effects of HLJDD should be studied using integrated omics technologies, including genomics, transcriptomics, proteomics, metabolomics, and radiomics, to broaden the understanding of the underlying mechanisms of HLJDD and its bioactive components. In addition, experimental and clinical studies on the hepatic and renal toxicity of HLJDD in ischemic stroke should be extensively conducted. In the future, well-designed clinical trials with long-term follow-ups should also be performed to evaluate therapeutic effects in post-stroke recovery.
